# The Therapeutic Potential of Antioxidants in the Prevention of Human Diseases

**DOI:** 10.3390/ijms26125891

**Published:** 2025-06-19

**Authors:** Kota V. Ramana

**Affiliations:** Department of Biomedical Sciences, Noorda College of Osteopathic Medicine, Provo, UT 84606, USA; karamana@noordacom.org

The recent surge in various chronic infectious and non-infectious diseases worldwide has created an urgent need for safe and effective prevention and treatment options to control and manage these diseases. For example, although the recent COVID-19 pandemic is now well under control, the emergence of various new variants still requires vigorous investigations and the development of new therapeutic strategies [1]. Increased oxidative stress has been associated with the pathogenesis of a wide array of human disorders, including cardiovascular diseases, cancer, neurodegenerative conditions, diabetes, and inflammatory disorders. Oxidative stress occurs due to an imbalance between the production of reactive oxygen species (ROS) and cellular antioxidant defense mechanisms. An increased number of ROS can damage cellular components such as DNA, proteins, and lipids. ROS can also alter various cellular metabolic and inflammatory signaling pathways involved in the pathophysiology of various diseases.

Several anti-inflammatory and anti-oxidative agents have shown potential in controlling and managing human disease complications. In the past, natural medical practices such as Ayurveda, Unani, and traditional Chinese medicine have been used, employing various plant-based treatments to control human diseases without knowing their molecular mechanisms of action. However, studies from the past few decades and recent ongoing studies have shown that natural antioxidants derived from various plants have the capacity to neutralize oxidative stress and the inflammatory signaling pathways responsible for multiple pathologies. Antioxidants, both endogenous, such as glutathione, superoxide dismutase, and catalase, and exogenous, such as vitamins and plant polyphenols, could play a critical role in maintaining redox homeostasis and protecting tissues from oxidative injury.

Further, several preclinical and clinical studies have identified the therapeutic potential of antioxidants [2,3,4,5]. The dietary intake of antioxidant-rich foods has been associated with a reduced risk of chronic diseases. Moreover, targeted antioxidant therapies using curcumin [6], benfotiamine [7], quercetin [8], and other polyphenols [9] have shown promising results in improving disease outcomes in animal models, and some of these agents have undergone clinical studies for multiple disease pathologies. Some studies have also investigated the use of antioxidants as adjunct therapies to improve drug resistance and the efficacy of the treatment, particularly in various cancer chemotherapies.

Despite recent advances, the translation of antioxidant therapy to the clinical setting remains complex and needs further investigation. The efficacy of antioxidants can depend on their dosage and bioavailability and the disease context. Further, the unnecessary and or excessive intake of antioxidants or vitamins could disrupt the physiological redox balance and redox signaling. This leads to increased innate immune and inflammatory responses and unintended consequences. Therefore, a better understanding of how antioxidants regulate redox signaling is critical for their therapeutic development.

The studies published in this Special Issue include a combination of cutting-edge research articles and comprehensive review articles to explore the role of antioxidants in disease prevention and treatment. From molecular mechanisms to clinical applications, these articles offer insights into how antioxidant strategies can mitigate oxidative stress and improve human health.

The first study by Jang et al. explores the potential of herbal medicine as a treatment for postmenopausal osteoporosis using multi-scale network and random-walk-based analyses. By prioritizing herbs based on their protein target relevance to osteoporosis, they have identified promising herb candidates for modulating various protein targets relevant to osteoporosis, such as *Benincasae Semen*, *Glehniae Radix*, and *Houttuyniae Herba*. This study also identified active compounds like falcarindiol and tetrahydrocoptisine, which could regulate inflammation and bone metabolism pathways, providing new insights into herbal strategies for managing osteoporosis.

The study by Jovanovic et al. evaluated the antidiabetic potential of xanthone-rich extracts from *Gentiana dinarica* and *Gentiana utriculosa plants.* These extracts were analyzed for their secondary metabolite content and assessed for their antioxidant activity, α-glucosidase inhibition, and anti-hyperglycemic effects in glucose-loaded Wistar rats. The findings from this study indicated that extracts with higher concentrations of specific xanthones, norswertianin, and decussatin derivatives exhibited the most substantial potential to control blood glucose levels and scavenge free radicals, thus supporting their therapeutic relevance to diabetes management. Further, the study by Fonseca et al. investigated the antifungal potential of caffeine against *Trichophyton mentagrophytes*, a common cause of skin infections. Caffeine exhibited significant anti-dermatophyte activity with a minimum inhibitory concentration (MIC) of 8 mM, disrupting the fungal cell wall’s components and ultrastructure and inducing autophagic-like changes. Caffeine also protects human keratinocytes during infection and inhibits fungal spore germination. These results suggest that caffeine is a potential therapeutic and preventive agent for dermatophytosis.

Another article by Hernandez et al. examined the effects of adding Coenzyme Q10 (CoQ10) to an antioxidant supplement (Nutrof Total^®,^ Thea Pharmaceuticals, Keele, UK) on human retinal cells under oxidative stress. The combined supplement (NQ) showed synergistic benefits by controlling oxidative stress and mitochondrial dysfunction. This study thus suggests that CoQ10 could be a promising therapeutic strategy for degenerative retinal diseases such as age-related macular degeneration and diabetic retinopathy.

The randomized crossover clinical trial study by Lambadiari et al. has reported on the effects of the Mediterranean (MD) and Ketogenic (KD) diets on patients with psoriasis (PSO) and psoriatic arthritis (PSA) over 22 weeks. Both diets significantly reduced weight, body mass index, waist circumference, total fat, and visceral fat. However, only the KD showed significant improvements in disease activity and inflammatory markers, suggesting it may be more effective in managing PSO and PSA symptoms.

Another interesting study by Sandoval et al. evaluated the effects of β-carotene on the pancreas of C57BL/6 mice administered with moderate amounts of ethanol. Mice exposed to moderate-dose alcohol who were treated with β-carotene showed significantly higher amylase and lower lipase levels and notable differences in pancreatic fibrosis and islet structure across groups. These results suggest that antioxidant treatments like β-carotene may help to mitigate ethanol-induced pancreatic tissue damage.

The review article by Frost et al. explored the significance of water-soluble B vitamins in cancer progression and prevention. The authors discussed the role of B vitamins in cancer, where they can support tumor suppression and, in some cases, promote cancer progression by influencing metabolic pathways like glycolysis and mitochondrial function. The authors also emphasized the need for further studies to better understand how to balance B vitamin intake to optimize cancer prevention and treatment outcomes. Flavonoids are known for their anti-inflammatory and antioxidant properties. Flavonoids have shown promise in improving liver health and modulating gut microbiota, a key factor in metabolic-associated fatty liver disease (MAFLD) progression. Another review by Sokal-Dembowska et al. discussed the current evidence on how specific flavonoids may help to prevent or slow MAFLD. Further, they elaborated on how flavonoids could be used in personalized dietary strategies for managing liver diseases. Finally, Jaiswal et al. discussed the various pharmacological activities of *Leopoldia comosa* (LC), a Mediterranean plant traditionally used in food and medicine. The authors briefly discussed LC’s multiple biological activities, such as its antioxidative, anti-inflammatory, and anti-carcinogenic effects, and outlined the gaps in the research into LC’s pharmacological effects and its therapeutic development.

Although recent growing evidence supports the therapeutic efficacy of natural antioxidants in the prevention and management of various human diseases, there are some significant gaps that need further investigation. One major gap is the inconsistency between preclinical in vitro or animal model findings when compared to human clinical outcomes. These studies sometimes show differences in bioavailability, dosage, and long-term effects. Additional clinical studies are necessary to address the variability in genetics, metabolism, and microbiota, which can influence antioxidant efficacy and safety profiles. Further, identifying standardized methodologies to evaluate antioxidant activity and limited large-scale, long-term clinical trials to confirm their preventive benefits across diverse populations could help advance antioxidant treatments to the clinical setting. Furthermore, novel targeted antioxidant delivery and personalized nutrition strategies could also help establish interventions for human diseases.
